# Unusual Case of Obstructive Jaundice

**DOI:** 10.7759/cureus.4094

**Published:** 2019-02-19

**Authors:** Rajesh Essrani, Eric Nellis, Patrick Hickey, Hiral Shah

**Affiliations:** 1 Internal Medicine, Lehigh Valley Health Network, Allentown, USA; 2 Gastroenterology, Lehigh Valley Health Network, Allentown, USA

**Keywords:** obstructive jaudice, head and neck cancer, duodenum

## Abstract

Most malignant obstructive jaundice arises from primary periampullary tumors and rarely from metastatic cancer of the head and neck. A 60-year-old male was diagnosed with obstructive jaundice due to metastatic squamous cell carcinoma of the tonsil. Only 12 cases of small bowel metastasis from the head and neck have been reported. Most of them originate from laryngeal squamous cell carcinoma, and only one case reported tonsillar cancer metastasizing to the ileum. Our case is the first one, to the best of our knowledge, to illustrate tonsillar cancer with metastasis to the duodenum causing obstructive jaundice.

## Introduction

Most malignant obstructive jaundice stems from primary periampullary tumors, such as the pancreas, duodenum, distal common bile duct, and structures of the ampullary complex. Small bowel metastases from the head and neck are very rare. Autopsy studies showed that the most common sites of metastases in head and neck cancer carcinoma are the lungs (72%), liver (38.6%), kidney (21%), adrenal (21%), bone (23%), and, rarely, the heart (12%) and small intestine (7%) [1]. The incidence of tonsillar cancer, a subtype of head and neck cancers has increased by four times in the United States over the last few decades due to human papillomavirus (HPV) infections and smoking [2]. Tonsillar carcinoma usually metastasizes locally to the cervical lymph nodes [3]. Early stage cancers rarely have distal metastasis but advanced stage cancers can metastasize beyond the cervical lymph nodes [4]. It spreads through the lymphatic and/or vascular channels [5]. We present an unusual case of obstructive jaundice secondary to metastatic tonsillar cancer to the duodenum.

Parts of this article are based on a poster: Rajesh Essrani MD, Eric Nellis MD, Patrick Hickey DO, Hiral Shah MD. Unusual Case of Obstructive Jaundice. World Congress of Gastroenterology at ACG; October 2017.

## Case presentation

A 60-year-old male with a remote history of tonsillar squamous cell cancer (SCC) treated with chemoradiation presented with a three-day history of new-onset epigastric pain radiating to his back, which was associated with nausea and vomiting. He had a remote smoking history but no alcohol intake. Three months before this presentation, he was found to have metastatic SCC in the jejunum, which was treated with curative resection.

**Figure 1:**
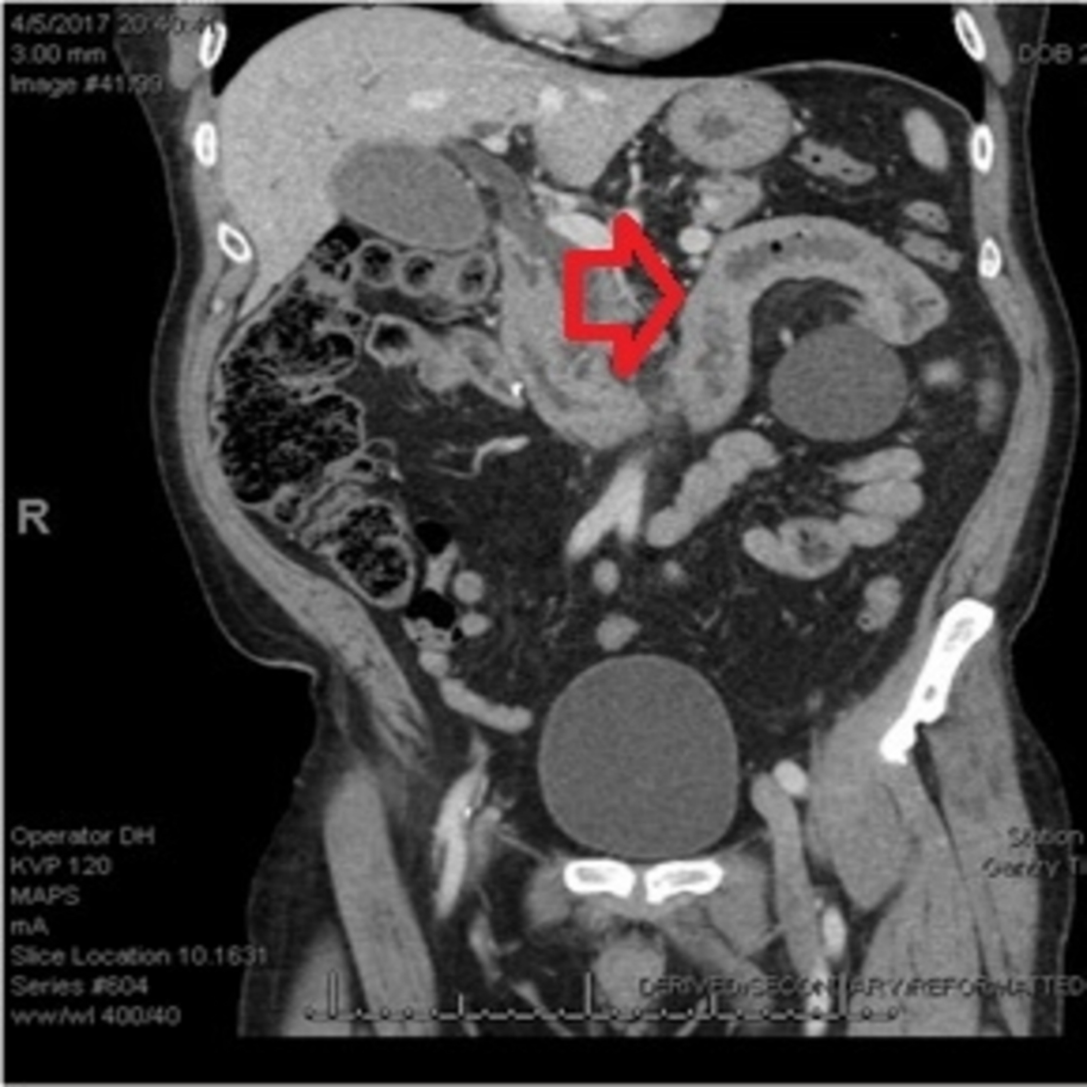
CT abdomen-pelvis showing thickening of the duodenum and proximal small bowel computed tomography (CT)

In addition to his severe pain, the patient noted a 14-pound weight loss over the past three weeks due to his symptoms of anorexia with nausea and vomiting. On clinical examination, vital signs were stable; icterus was present. Abdominal exam was very tender to palpitation in the epigastric region but with normal bowel sounds. He had multiple abnormalities in his liver function panel, including aspartate aminotransferase (AST) - 160 U/L, alanine aminotransferase (ALT) - 218 U/L, alkaline phosphatase - 281 U/L, and total bilirubin - 3.0 mg/dl. Lipase was also markedly elevated at 10304 U/L. Right upper quadrant ultrasound showed biliary sludge. Computed tomography (CT) abdomen with contrast showed gallbladder distention and mild prominence of the intra-and extrahepatic bile duct (Figure 1).

**Figure 2:**
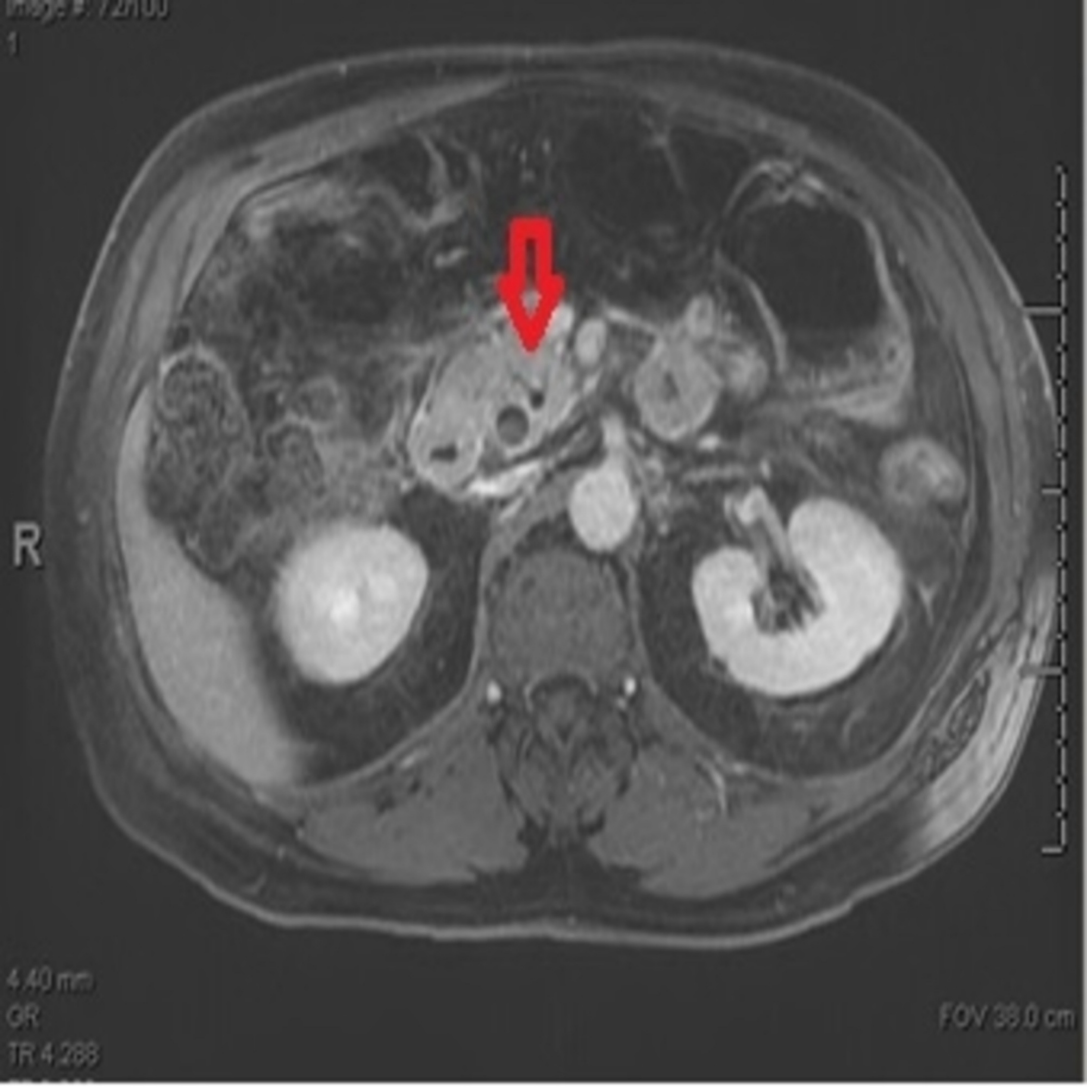
MRI abdomen showing double duct sign magnetic resonance imaging (MRI)

Magnetic resonance imaging (MRI) abdomen showed a double duct sign (Figure 2).

**Figure 3:**
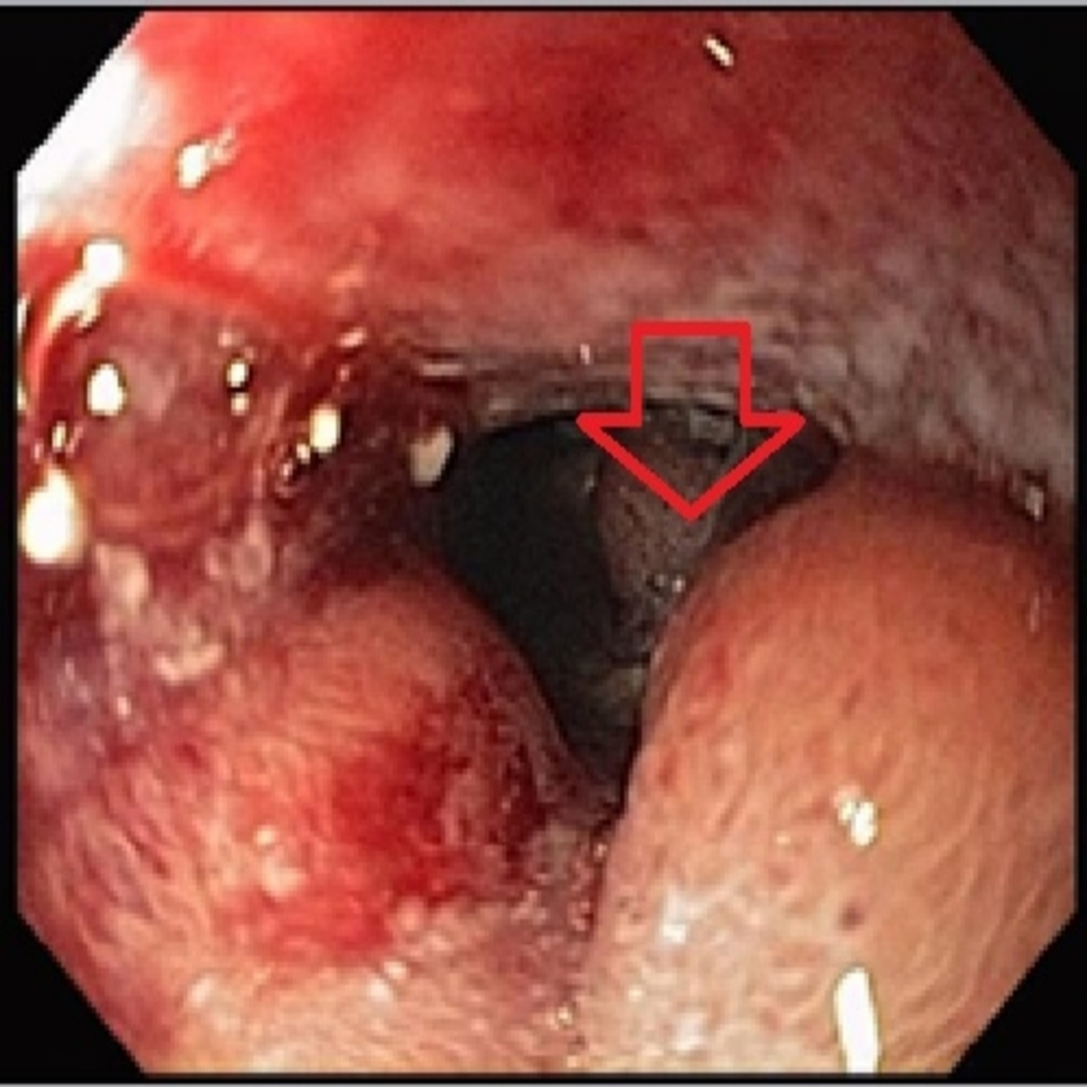
EGD showing infiltrative thickening of the duodenal bulb esophagogastroduodenoscopy (EGD)

He underwent esophagogastroduodenoscopy (EGD), which showed infiltrative thickening of the duodenal bulb and the second and third portions of the duodenum (Figure 3).

Multiple biopsies were taken, and he underwent endoscopic retrograde cholangiopancreatography (ERCP) with biliary stent and drain placement. His abdominal pain improved significantly and was discharged in a stable condition. Pathology of the biopsied mass was consistent with metastatic squamous cell carcinoma of the tonsil.

## Discussion

Obstructive jaundice can be caused by a variety of etiologies, which include either primary or metastatic neoplasm, choledocholithiasis, biliary strictures, parasites, primary sclerosing cholangitis, and cystic duct stones.

Head and neck cancer comprise a variety of cancers with squamous cell carcinoma (HNSCC) being the most common. Risk factors for cancer include smoking, HPV infection, betel nut chewing, radiation exposure, vitamin deficiencies, periodontal disease, immunosuppression, and other environmental and occupational exposures. The clinical presentation depends upon the primary site, distant metastatic site, and exposure to various risk factors. Small bowel metastasis from HNSCC, as any other SCC distant metastasis, results from hematogenous dissemination of the disease and represents florid micrometastasis from the primary tumor [6].

The most common presentation of the head and neck cancer metastasizing to the small bowel is gastrointestinal bleeding, perforation, and gastric outlet obstructive symptoms but, rarely, they might develop obstructive jaundice.

This patient had a history of tonsillar SCC with metastasis to the jejunum, so suspicion was very high for SCC metastasis. Only 12 cases of small bowel metastasis from the head and neck have been reported. Most of them originate from laryngeal SCC, and only one case reported tonsillar cancer metastasizing to the ileum [7]. Our case is the first one to illustrate tonsillar cancer with metastasis to the duodenum causing obstructive jaundice [8]. Diagnosis is made through endoscopic biopsy.

## Conclusions

During a review of the literature, no previous case reporting tonsillar cancer with metastasis to the duodenum causing obstructive jaundice was found. The diagnosis was made through an endoscopic biopsy. A patient with an obstructive jaundice feature and a history of cancer should raise the suspicion of metastatic cancer and should receive appropriate workup and treatment.
